# Comparative Evaluation of Incidence of Cardiovascular Events Among Different Drug‐Eluting Stent Features: A Retrospective Cohort Study

**DOI:** 10.1002/hsr2.71287

**Published:** 2025-09-23

**Authors:** Hossein Koushki, Reza Golchin Vafa, Reza Heydarzadeh, Houyar Zarifkar, Amin Khadem Hosseini, Houman Zarifkar, Hourshad Zarifkar, Alireza Azadian, Farhang Amiri, Ali Mohammadhassani, Mohammad Montaseri, Nazanin Hosseini, Mehrdad Sadeghi, Seyed Ali Hosseini, Seyed Alireza Mirhosseini, Javad Kojuri

**Affiliations:** ^1^ Shiraz University of Medical Sciences Shiraz Iran; ^2^ Cardiology Department Shiraz University of Medical Sciences Shiraz Iran; ^3^ Professor Kojuri Cardiology Clinic Niayesh Medical Complex Shiraz Iran; ^4^ School of Medicine, Cardiovascular Research Center Shiraz University of Medical Sciences Shiraz Iran; ^5^ Clinical Education Research Center Shiraz University of Medical Sciences Shiraz Iran

**Keywords:** drug‐eluting stents, major adverse cardiovascular events, percutaneous coronary intervention

## Abstract

**Background and Aims:**

Coronary artery disease (CAD) is often treated with percutaneous coronary intervention (PCI). Various stent types are used during PCI, potentially resulting in different outcomes. Here, we compared major adverse cardiovascular events (MACE) across different stents over a 5‐year follow‐up post‐PCI.

**Methods:**

This retrospective cohort study analyzed individuals receiving PCI from Iranian databases. Inclusion criteria encompassed drug‐eluting stent recipients, excluding emergency surgical cases. Demographics, medical history, angiography, PCI details, and follow‐up on MACE were collected and analyzed using Cox regression analyses.

**Results:**

Out of 4159 recruited patients, MACE were observed in 10.2% of individuals over 5 years. Factors significantly associated with MACE included a history of heart failure (HR: 2.58, 95% CI: 1.92–3.47), hypertension (HR: 1.87, 95% CI: 1.52–2.30), and lower baseline ejection fraction (HR: 0.975, 95% CI: 0.952–0.997). Procedural elements like the number of stents used (HR: 1.008, 95% CI: 1.005–1.012) and their total length (HR: 1.008, 95% CI: 1.005–1.012) were also significant predictors. No significant relationship was found between the types of drug‐eluting stents and MACE.

**Conclusion:**

In a 5‐year study of post‐PCI patients, MACE rates were higher in those with lower ejection fractions and those with more stented vessels and longer stents but similar across different drug‐eluting stents.

**Abbreviations**
AES: amphilimus‐eluting stent
AHA: American Heart Association
BES: biolimus‐eluting stent
BMI: body mass index
BMS: bare‐metal stents
BVS: bioresorbable vascular scaffold
CABG: coronary artery bypass graft
CAD: coronary artery disease
CI: confidence interval
CKD: chronic kidney disease
CVA: cerebrovascular accident
CVD: cardiovascular disease
DBP: diastolic blood pressure
DES: drug‐eluting stents
DHF: decompensated heart failure
DM: diabetes mellitus
EES: everolimus‐eluting stent
EF: ejection fraction
HLP: hyperlipidemia
HR: hazard ratio
HTN: hypertension
ISR: intracoronary stent restenosis
LAD: left anterior descending artery
LCX: left circumflex artery
MACE: major adverse cardiovascular events
PCI: percutaneous coronary intervention
PC‐DES: polymer‐coated DES
PES: paclitaxel‐eluting stents
PF‐DES: polymer‐free DES
RCA: right coronary artery
SBP: systolic blood pressure
SES: sirolimus‐eluting stents
ZES: zotarolimus‐eluting stent

## 1 Introduction

Cardiovascular disease (CVD) is the primary cause of death globally. This disease is progressive, and its prevalence has significantly increased in recent decades [1]. Coronary artery disease (CAD) is the most common type of CVD, characterized by decreased blood flow to the cardiac muscles, mainly due to atherosclerosis. Atherosclerosis begins with inflammation and progresses with fat deposition in vessel walls [2, 3]. Various strategies exist for treating CAD. While some patients require medication to slow its progression, others may need more invasive procedures. Medications such as antiplatelets, cholesterol‐lowering agents, and beta‐blockers are available for CAD management [4]. Coronary artery bypass grafting (CABG) is typically reserved for those with complex CAD, multiple blocked vessels, and left main disease [4, 5]. Percutaneous coronary intervention (PCI) is now preferred over CABG for patients with significant CAD due to its reduced invasiveness and shorter recovery time [6]. PCI complications include hemorrhage, arterial perforation, thrombosis, and major adverse cardiovascular events (MACE) [6, 7, 8]. MACE lacks a specific definition but primarily encompasses cardiac death, myocardial infarction (MI), stroke, revascularization, and rehospitalization due to CVD [8].

The number and type of stents are crucial factors influencing MACE. Three main stent types have been utilized in PCI, including bare‐metal stents (BMS), drug‐eluting stents (DES), and bioresorbable vascular scaffolds (BVS). BMS results in intracoronary stent restenosis (ISR) in 10%–20% of patients after a 6‐month follow‐up [6]. DES are modern types of stents with three generations, used to inhibit neointimal hyperplasia and reduce ISR compared to BMS. Different generations vary based on materials and surface coating. In first‐generation DESs like sirolimus‐eluting stents (SES) and paclitaxel‐eluting stents (PES), the coating is nondegradable; however, in second‐generation DESs like the everolimus‐eluting stent (EES) and zotarolimus‐eluting stent (ZES), the coating is degradable [6, 9].

Several studies have demonstrated varying MACE prevalence among different DES. One study observed higher MACE prevalence in PES compared to SES after 1‐year follow‐up but no significant difference after 3 years [10]. Another study indicated better outcomes after 2 years of follow‐up for patients with SES than ZES or PES. However, other studies found no differences between different DES and cardiovascular outcomes [11]. Given the conflicting findings in the literature regarding the impact of various DESs on MACE prevalence, we assessed the effects of different stent features on MACE occurrence during a 5‐year study of post‐PCI individuals.

## 2 Methods

This retrospective cohort study involved 4438 individuals who received PCI from March 2018 to February 2020. Data were obtained from the Iranian Network of Cardiovascular Research Registry (which registered post‐PCI patients all around Iran in more than 40 public and private hospitals) and the database of Professor Kojuri Cardiology Clinic (Niyayesh St., Shiraz, Iran, www.kojuriclinic.com). After each procedure, expert cardiologists recorded baseline demographic data, prior medical history, angiography, and PCI reports.

### 2.1 Inclusion and Exclusion Criteria

**Figure 1:**
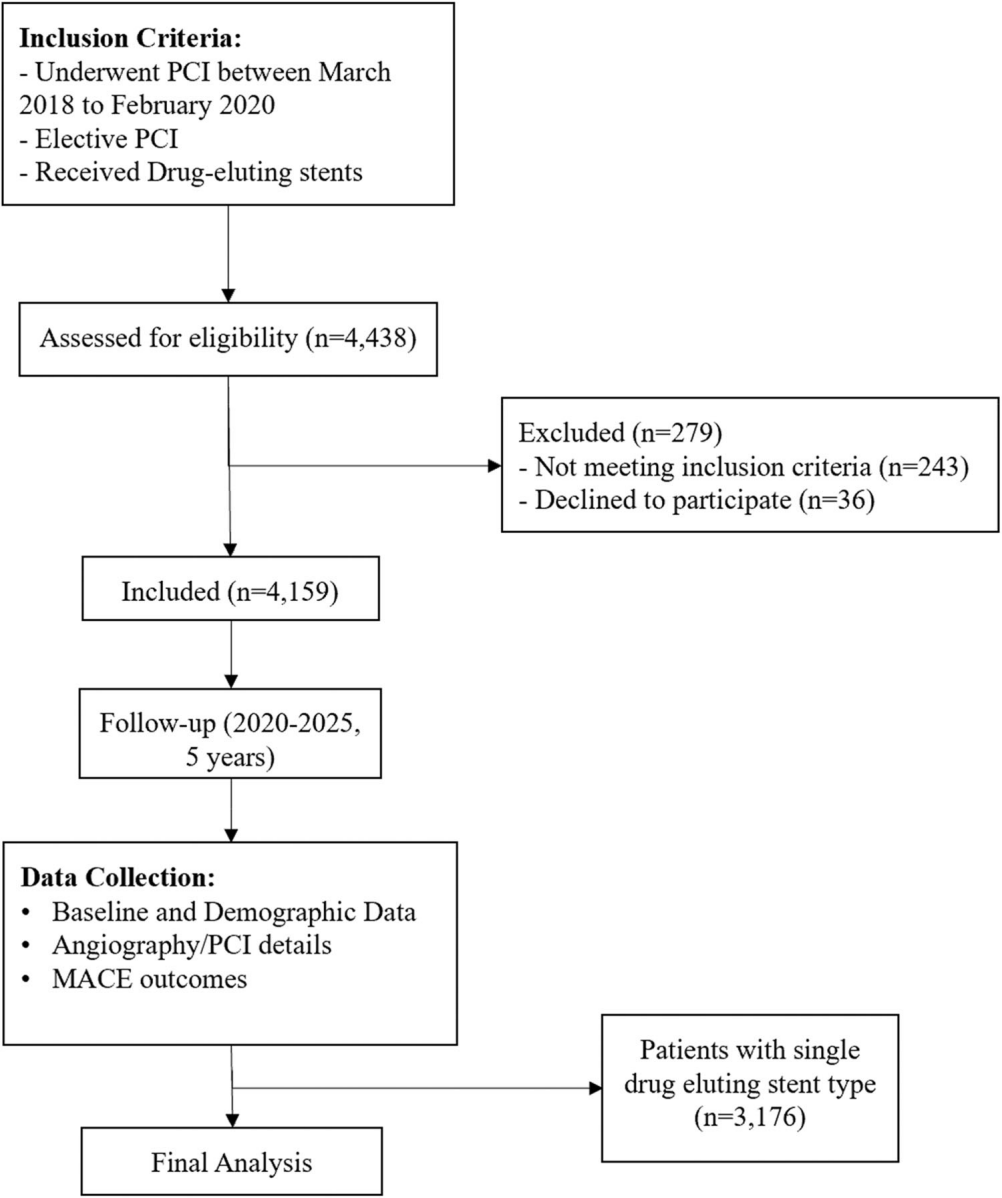
Study flow diagram.

We included participants who underwent elective PCI and received DES insertion. Individuals with more than one type of DES were excluded. Patients who underwent emergent CABG after PCI were excluded (Figure 1).

### 2.2 Demographics and Baseline Data

Demographic data including age, gender, body mass index (BMI), family history of CAD, prior CAD history, hypertension (HTN), diabetes mellitus (DM), hyperlipidemia (HLP), and kidney diseases were obtained. We also collected data on the prevalence of cigarette smoking, opium addiction, and alcohol addiction. Additionally, information on clinical manifestations of the disease, including stable angina, unstable angina, ST‐segment elevation MI, and non‐ST‐segment elevation MI, was extracted.

According to the American Heart Association (AHA), Stage 1 HTN is defined as a systolic blood pressure of 130–139 mmHg or a diastolic blood pressure of 80–89 mmHg [12]; we defined HTN as Stage 1 or higher blood pressure. DM was defined according to the American Diabetes Association as having a hemoglobin A1c level of at least 6.5%, a fasting plasma glucose level of 126 mg/dL or higher, an oral glucose tolerance test level of 200 mg/dL or greater, or a random plasma glucose level of 200 mg/dL or above [13]. Based on the 2019 AHA guideline, a low‐density lipoprotein cholesterol level exceeding 160 mg/dL or a nonhigh‐density lipoprotein cholesterol over 190 mg/dL was considered as HLP [14]. Smoking status was categorized as: (1) current smokers (active tobacco use within the past 3 months), (2) recent smokers (cessation > 3 months prior), and (3) never smokers (no lifetime tobacco use). Current smokers and former smokers with ≤ 1 month of smoking cessation were considered current smokers [15].

### 2.3 Angiography and PCI Data

We collected the angiography and PCI data, including the type and location of coronary artery lesions and the type, location, size, diameter, and number of stents. DES encompasses a variety of types, including the biolimus‐eluting stent (BES), amphilimus‐eluting stent (AES), SES, ZES, PES, and EES. Within the BES category, a notable example is the BioMatrix stent, while SES includes options like BioMime, Orsiro, Cre8, SUPRAFLEX, ULTIMASTER, and Coroflex ISAR. ZES variants include the Resolute Onyx and Resolute Integrity stents, whereas EES comprises options such as Promus PREMIER, Promus Element, and XIENCE. Polymer‐coated drug‐eluting stents (PC‐DES) include BioMatrix, BioMime, Orsiro, SUPRAFLEX, ULTIMASTER, Resolute Onyx, Resolute Integrity, Promus PREMIER, Promus Element, and XIENCE. Conversely, polymer‐free drug‐eluting stents (PF‐DES) are exemplified by Cre8 and Coroflex ISAR. Regarding generational classification, the first generation includes BioMime, Orsiro, Cre8, SUPRAFLEX, ULTIMASTER, and Coroflex ISAR. Second‐generation members are Promus PREMIER, Promus Element, and XIENCE, alongside Resolute Onyx and Resolute Integrity. Finally, the BioMatrix stent represents the third generation.

We also extracted information about ballooning, including predilatation, postdilatation, and the number of balloons used. We extracted data about different PCI techniques, including kissing and overlapping. We classified coronary legions according to the ACC/AHA classification: Type A: < 10 mm, nonangulated, smooth, little calcification, not totally occlusive, not ostial, no major branch involvement, no thrombus; Type B: 10–20 mm, eccentric, moderately tortuous, 45°–90°, irregular, moderate to heavy calcification, ostial, bifurcation lesions, some thrombus (subcategories: B1: one characteristic; B2: two or more characteristics), and Type C: diffuse, extremely angulated, > 90°, inability to protect major side branch, degenerated vein graft [16].

### 2.4 Follow‐Up

The study enrolled patients undergoing PCI between March 2018 and February 2020 (a 24‐month enrollment period) with planned follow‐up extending up to 5 years postprocedure (through February 2025). All patients received standardized antiplatelet therapy according to contemporary PCI guidelines. Dual antiplatelet therapy was initiated immediately postprocedure, consisting of aspirin (80 daily) plus a P2Y12 inhibitor, clopidogrel (75 mg daily) or ticagrelor (90 mg twice daily) based on physician judgment and patient characteristics. This regimen was maintained for 12 months, after which patients transitioned to aspirin monotherapy (80 mg daily). Adherence was monitored through: (1) structured patient interviews during follow‐up visits (1, 3, 6, and 12 months), (2) verification of prescription refills via pharmacy records, and (3) documentation of therapy discontinuation or changes in the medical record.

MACE was assessed following PCI, which includes MI, stroke, heart failure, and cardiac death, as well as any revascularization. For the primary MACE analysis, revascularizations were included within MI outcomes when concurrent to avoid duplicate counting. All‐cause mortality explicitly includes both cardiac and noncardiac deaths. All events were adjudicated by a team of cardiology residents outside of the research team. Additionally, the date of MACE occurrence after PCI was recorded, and information on noncardiac‐related deaths was collected.

### 2.5 Data Analysis

We analyzed the data using SPSS (IBM Corp. Released 2019. IBM SPSS Statistics for Windows, Version 26.0. Armonk, NY). Continuous data are expressed as mean ± standard deviation (SD), while categorical variables are presented as frequency and percentage. We used independent sample *t*‐test and Pearson's *χ* ^2^ test to compare variables between groups. For variables with more than two categories, we employed one‐way ANOVA. Additionally, we utilized Cox regression to analyze the effects of different predictors on the incidence of the outcome. *p* values < 0.05 were considered significant.

To identify predictors of MACE, we conducted both univariable and multivariable Cox proportional hazards regression analyses. In the univariable analysis, all candidate predictors—including demographic characteristics, comorbidities, procedural variables, and stent‐related factors—were individually evaluated. These included demographic factors (age, sex), comorbidities (DM, HTN, chronic kidney disease [CKD]), procedural characteristics (number of stents, total stent length), and stent‐related factors (type, generation, polymer coating). Variables with clinical relevance or a univariable *p* < 0.10 were subsequently entered into the multivariable model to adjust for potential confounding. The final model reported HRs with 95% confidence intervals (CIs). All statistical tests were two‐sided, and significance was set at *p* < 0.05.

Known confounders—including prior PCI, number of stents implanted, and total stent length—were included in the multivariable model regardless of univariable significance due to their established influence on cardiovascular outcomes. Variables were excluded from the multivariable model if they showed high collinearity with other variables (variance inflation factor > 5) or had more than 10% missing data, which could introduce bias or instability into the estimates. These exclusions were documented and discussed as study limitations.

### 2.6 Ethical Considerations

Patients were informed about the research details and informed consent was obtained. Their information remained confidential. The Shiraz University of Medical Sciences Ethical Committee approved the study protocol under the Ethics Code IR.SUMS.MED.RED.1402.409.

## 3 Results

### 3.1 Baseline Data and Demographics

A total of 4438 Iranian subjects were considered for the study, but 279 were not included due to incomplete data, refusal to consent, or an inability to follow‐up (Figure 1). Out of the 4159 patients included in the study, there were 1255 women (30.2%) and 2904 men (69.8%). The mean age of the participants was 63.7 ± 12.6 years. The mean BMI was 26 ± 3.7 kg/m^2^. Regarding cardiovascular risk factors, 938 had a family history (22.6%) of CVD, 2789 had HTN (67.1%), 1449 had DM (34.8%), and 2355 had HLP (56.6%). There were 522 current smokers (12.6%) and 189 recent smokers (4.5%). The prevalence of alcohol and opium addiction was 17 (0.4%) and 222 (5.3%), respectively (Table S1).

The occurrence of previous PCI and CABG procedures was 627 (15.1%) and 227 (5.5%), respectively. Peripheral artery disease was reported in only five patients (0.1%). Additionally, 38 patients (0.9%) were diagnosed with heart failure. The mean ejection fraction (EF) before the PCI was 50.3% ± 10.8%. CKD was present in 62 (1.5%) patients. The prevalence of stable and unstable angina was 2400 (57.7%) and 610 (14.7%), respectively. Moreover, 369 participants presented with ST‐segment elevation MI, while 331 patients (8%) had non‐ST‐segment elevation MI (Table S1).

### 3.2 Angiography

The prevalence of one‐vessel, two‐vessel, and three‐vessel disease based on the angiography was 2532 (60.9%), 1323 (31.8%), and 304 (7.3%) counts, respectively. Moreover, 1703 (40.9%) patients had only one coronary lesion, 1274 (30.6%) people had two coronary lesions, and 1182 (28.4%) had three or more than three coronary lesions. The most common lesion was left anterior descending artery (LAD) lesions (2951, 71%). The prevalence of left circumflex artery (LCX) and right coronary artery (RCA) lesions was 1536 (36.9%) and 1603 (38.5) cases, respectively. There were 2657 proximal (63.9%), 2029 midpart (48.8%), and 599 distal (14.4%) lesions. In total, 512 Type A (12.3%), 1738 Type B1 (41.8%), 1631 Type B2 (39.2%), and 1775 Type C (42.7%) lesions were seen on the angiography (Table S2).

### 3.3 PCI

Balloon PCI was performed on 3951 (95%) of patients, including 3404 (81.8%) who underwent predilatation, 2403 (57.8%) who had postdilatation, and 216 (5.2%) who received kissing balloon PCI. In addition, 1938 patients (46.6%) received one coronary stent, 1373 (33%) received two coronary stents, and 848 patients (20.3%) had three or more stents. The average total stent length per patient was 50.6 ± 33.2 mm. The mean stent diameter was 3.2 ± 1.1 mm. Furthermore, 4133 patients (99.4%) received PC‐DESs, 8 (0.1%) received PF‐DESs, and 18 (0.4%) received both polymer and polymer‐free stents. Additionally, 3178 patients (76.4%) received one type of DES, 960 (23.1%) received two types, and 21 (0.5%) received three types. The prevalence of different types of DESs was 2543 (61.2%) EES, 2434 (61.1%) SES, 83 (2%) ZES, 79 (1.9%) BES, 2 (< 0.01%) AES, and 1 (< 0.01%) PES (Table S3).

### 3.4 MACE and All‐Cause Mortality

After a 5‐year study, 426 (10.2%) MACE were recorded. The average time to develop a MACE after PCI was 603 ± 400 days. We identified 278 (6.7%) new MI, 25 (0.6%) cerebrovascular accident (CVA), 42 (1%) decompensated heart failure (DHF), and 81 (1.9%) cardiac death occurrences. Noncardiac causes contributed to 84 (2%) of the total mortality cases. Patients who received three types of DESs had a significantly higher occurrence of MACE (7, 33%) compared to those with two (135, 14.1%) or one type of DESs (284, 8.9%) (*p* = 0.001). After adjusting for the number of stents used and total stent length per patient, there were also significant differences between the occurrence of MACE and the number of types of DESs used (*p* = 0.04). However, no significant differences were observed between noncardiac deaths and the number of types of DESs used (*p* = 0.177).

The data of 4159 patients were extracted and described. However, the data of 3176 patients who had received a single type of DES were analyzed for MACE occurrence and all‐cause mortality (Figure 1). Patients with AES or PES stents (*n* = 2) were excluded due to their rarity. This exclusion affected < 0.1% of the cohort and showed no significant distributional differences in baseline characteristics (all *p* > 0.20 via *χ* ^2^ tests), minimizing potential impact on overall conclusions. The prevalence of baseline characteristics and different risk factors among 3178 patients who received only one type of DES is shown in Table 1. Among patients who received one type of DES, the prevalence of MACE was 135 (9%) in SES, 145 (9%) in EES, 2 (5.9%) in ZES, and 2 (9.1%) in BES (Table 2). There were no significant differences between the occurrence of MACE, cardiac death, or all‐cause death and the DES used.

**Table 1:** Baseline characteristics of participants with different types of drug‐eluting stents.

		SES (*n* = 1507)	EES (*n* = 1613)	ZES (*n* = 34)	BES (*n* = 22)	*p*
Age (years)		65 ± 15	63 ± 10	59 ± 11	66 ± 8	0.170
Gender (male)		1063 (70.53)	1088 (67.45)	25 (73.53)	16 (72.73)	0.311
BMI (kg/m^2^)		26.09 ± 3.93	26.1 ± 3.74	25.1 ± 3.65	27.29 ± 3.62	0.134
Hypertension (*n*, %)		1013 (67.2)	1085 (67.26)	22 (64.71)	12 (54.55)	0.582
Diabetes mellitus (*n*, %)		497 (32.97)	605 (37.50)	8 (23.53)	5 (22.73)	**0.002**
Dyslipidemia (*n*, %)		871 (57.79)	917 (56.85)	18 (52.94)	11 (50)	0.407
Smoking (*n*, %)	Recent	83 (5.5)	59 (3.65)	2 (5.88)	1 (4.55)	**0.003**
	Current	180 (11.94)	188 (11.65)	2 (5.88)	3 (13.64)	
Alcohol addiction (*n*, %)		8 (0.53)	4 (0.24)	1 (2.94)	0 (0)	**0.001**
Opium addiction (*n*, %)		75 (4.97)	84 (5.20)	1 (2.94)	2 (9.09)	0.745
Peripheral arterial disease (*n*, %)		2 (0.13)	1 (0.061)	0 (0)	0 (0)	0.968
Chronic kidney disease		16 (1.1)	30 (1.85)	1 (2.94)	0 (0)	0.330
Heart failure (*n*, %)		15 (1.06)	16 (0.99)	0 (0)	0 (0)	0.901
Ejection fraction (%)		51 ± 11	50 ± 11	50 ± 10	30 ± 7	0.135
Positive family history (*n*, %)		397 (26.34)	291 (18.04)	5 (14.71)	7 (31.82)	**0.001**
Prior CABG (*n*, %)		91 (6.03)	82 (5.08)	2 (5.88)	2 (9.09)	0.196
Prior PCI (*n*, %)		222 (14.73)	228 (14.13)	0 (0)	6 (27.27)	**0.010**
Total stent length (mm)		41 ± 23	46 ± 29	35 ± 16	48 ± 28	**< 0.001**
Stent diameter (mm)		3 ± 1	3 ± 1	4 ± 2	4 ± 2	**< 0.001**
Total stents (*n*, %)	One	969 (64.2)	921 (57.1)	31 (94.1)	14 (63.6)	**0.001**
	Two	410 (27.2)	491 (30.4)	1 (2.9)	7 (31.8)	
	Three and more	128 (8.49)	201 (12.4)	3(8.8)	1 (4.5)	
Stable angina (*n*, %)		888 (58.92)	910 (56.41)	22 (64.71)	11 (50)	**0.001**
Unstable angina (*n*, %)		179 (11.87)	266 (16.49)	3 (8.82)	2 (9.09)	**0.001**
ST‐segment elevation MI (*n*, %)		101 (6.7)	147 (9.11)	4 (11.76)	0 (0)	**0.061**
Non‐ST‐segment elevation MI (*n*, %)		102 (6.76)	131 (8.12)	1 (2.94)	1 (4.55)	0.300

*Note:* All significant *p* values were shown in boldface.

Abbreviations: BES, biolimus‐eluting stent; BMI, body mass index; CABG, coronary artery bypass grafting; EES, everolimus‐eluting stent; EF, ejection fraction; MI, myocardial infarction; PCI, percutaneous coronary intervention; SD, standard deviation; SES, sirolimus‐eluting stent; ZES, zotarolimus‐eluting stent.

**Table 2:** The prevalence of major adverse cardiovascular events (MACEs) and all‐cause mortality in patients with different drug‐eluting stents.

	SES (*n* = 1507)	EES (*n* = 1613)	ZES (*n* = 34)	BES (*n* = 22)	*p*
Overall MACE	135 (8.95)	145 (8.98)	2 (5.88)	2 (9.09)	0.982
Myocardial infarction	79 (5.24)	94 (5.82)	1 (2.94)	1 (4.54)	
Cerebrovascular accident	15 (0.99)	8 (0.49)	0	0	
Heart failure	15 (0.99)	16 (0.99)	0	0	
Cardiac death	27 (1.79)	27 (1.67)	1 (2.94)	1 (4.54)	0.85
All‐cause mortality	35 (2.32)	35 (2.16)	0	0	0.63

Abbreviations: BES, biolimus‐eluting stent; EES, everolimus‐eluting stent; MACE, major adverse cardiovascular events; SES, sirolimus‐eluting stent; ZES, zotarolimus‐eluting stent.

**Figure 2:**
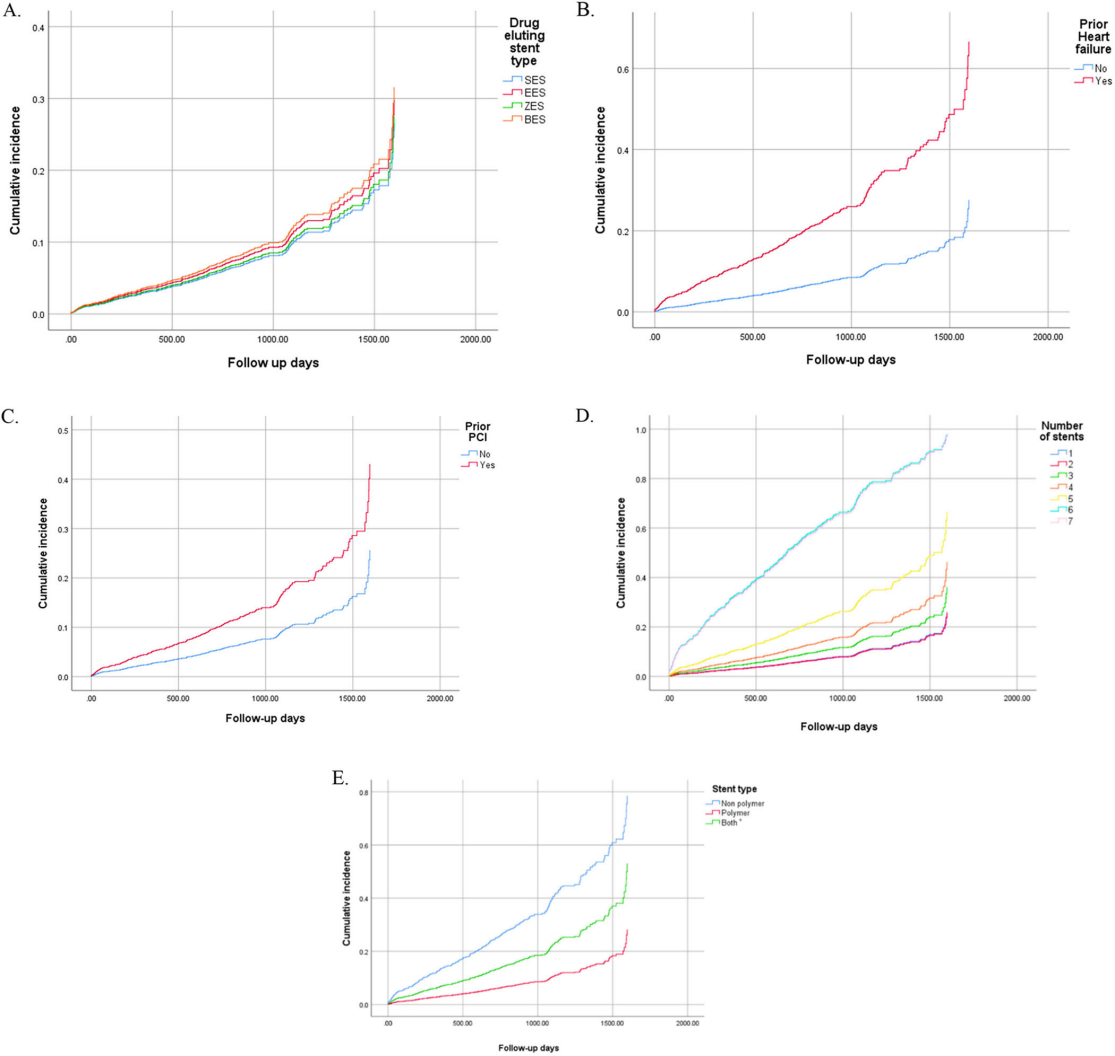
The cumulative incidence of major adverse cardiovascular events in (A) different groups of drug‐eluting stents, (B) patients with or without prior heart failure, (C) patients with or without prior percutaneous coronary intervention (PCI), (D) patients with different numbers of stents, (E) patients with polymeric or nonpolymeric stents. BES, biolimus‐eluting stent; EES, everolimus‐eluting stent; SES, sirolimus‐eluting stent; ZES, zotarolimus‐eluting stent. *Patients with both polymer and nonpolymer stents.

As presented in Figure 2 and Table 3, We investigated the impact of various factors on the occurrence of MACE. There were no significant differences between MACE and the type of DES. MACE was notably more common in patients with a history of heart failure (HR: 3.394, 95% CI: 1.852, 6.218, *p* < 0.001) and PCI (HR: 1.901, 95% CI: 1.454–2.485, *p* < 0.001). History of HTN had a borderline significant effect on the occurrence of MACE (HR: 1.295, 95% CI: 0.999, 1.677, *p* = 0.05). Patients with higher baseline EF experienced a lower rate of MACE (HR: 0.975, 95% CI: 0.952, 0.997, *p* = 0.028). The occurrence of MACE was greater in patients with a longer total stent length (HR: 1.008, 95% CI: 1.005, 1.012) and a greater number of stents. Patients with polymer stents had a lower occurrence of MACE than those with nonpolymer stents. DM and CKD did not significantly predict MACE within 5 years. The occurrence of MACE was also similar between different generations of DES. The occurrence of MACE was higher in nonpolymer stents (3, 37.5%) compared to polymer stents (418, 10.1%) (*p* = 0.002).

**Table 3:** Different predictors of major adverse cardiovascular events (MACE) in individuals with one type of drug‐eluting stent.

		*p*	Hazard ratio (95% CI)	*p* (between groups)
Age (years)		0.370	1.005 (0.994, 1.017)	
Male		0.879	1.020 (0.791, 1.315)	
Body mass index (kg/m^2^)		0.398	1.018 (0.977, 1.060)	
Alcohol consumption		0.797	0.773 (0.108, 5.512)	
Chronic kidney disease		0.119	1.698 (0.873, 3.304)	
Diabetes mellitus		0.076	1.240 (0.977, 1.572)	
Dyslipidemia		0.598	1.066 (0.841, 1.350)	
Heart failure		**< 0.001**	3.394 (1.852, 6.218)	
Hypertension		**0.05**	1.295 (0.999, 1.677)	
Baseline ejection fraction		**0.028**	0.975 (0.952, 0.997)	
Non‐ST elevation myocardial infarction		0.395	0.813 (0.504, 1.311)	
Opium addiction		0.302	1.394 (0.741, 2.620)	
Peripheral arterial disease		1		
Positive family history		0.076	1.268 (0.976, 1.647)	
Prior CABG		0.390	1.234 (0.775, 1.966)	
Prior PCI		**< 0.001**	1.901 (1.454, 2.485)	**< 0.001**
Smoking	No smoking		1 (reference)	
	Recent	0.969	1.037 (0.729, 1.474)	0.840
	Current		0.965 (0.572, 1.628)	0.894
Stable angina		0.542	0.929 (0.734, 1.176)	
ST‐segment elevation myocardial infarction		0.542	1.010 (0.664, 1.535)	
Unstable angina		0.155	1.253 (0.919, 1.710)	
Total stent length		**< 0.001**	1.008 (1.005, 1.012)	
Stent diameter		0.661	1.022 (0.928, 1.124)	
Polymer vs. nonpolymer stents	Nonpolymer	**0.014**	1 (reference)	
	Polymer		0.216 (0.069, 0.657)	**0.008**
	Both		0.493 (0.051, 4.742)	0.540
Number of stents	1	**< 0.001**	1 (reference)	
	2		0.977 (0.741, 1.290)	0.872
	3		1.481 (0.996, 2.202)	**0.052**
	4		2.052 (1.186, 3.549)	**0.01**
	5		3.617 (1.600, 8.179)	**0.002**
	6		12.987 (4.138, 40.765)	< **0.001**
	7		12.748 (1.778, 91.39)	**0.011**
Generation DES	First	0.5	1 (reference)	
	Second		1.149 (0.909, 1.453)	0.246
	Third		1.233 (0.305, 4.983)	0.769
Drug‐eluting stent type	SES	0.705	1 (reference)	
	EES		1.151 (0.909, 1.456)	0.243
	ZES		1.049 (0.259, 4.242)	0.946
	BES		1.233 (0.305, 4.982)	0.769

*Note:* All significant *p* values were shown in boldface.

Abbreviations: BMI, body mass index; CABG, coronary artery bypass grafting; CI, confidence interval; DES, drug‐eluting stent; PCI, percutaneous coronary intervention.

In the Cox regression model, prior PCI (HR: 1.90, 95% CI: 1.45–2.49), total stent length (HR: 1.01, 95% CI: 1.01–1.01), and heart failure (HR: 3.39, 95% CI: 1.85–6.22) were independent predictors of MACE. Variables such as stent diameter and medication history were excluded due to collinearity or missing data, respectively. Sensitivity analyses confirmed model robustness when including/excluding these variables.

## 4 Discussions

The principal finding of this study is that, over a 5‐year investigation, the type of DES used, whether based on generation or specific subtype, was not significantly associated with MACE or all‐cause mortality among patients who received a single type of DES. Despite the widespread use of various DES types, the incidence of MACE remained comparable across groups. Importantly, the data revealed that clinical factors such as a history of heart failure, prior PCI, lower baseline EF, and total stent length were more predictive of MACE than the specific DES type. These findings suggest that patient‐related risk factors and procedural characteristics may play a more critical role in long‐term outcomes than the stent type itself.

Numerous investigations in the literature have explored the impact of various DES on the occurrence of MACE across diverse follow‐up durations. Jensen and colleagues' study revealed that during the initial year postimplantation, the occurrence rate of MACE did not exhibit significant variance with EES compared to SES, but throughout 1–5 years, the occurrence of MACE notably decreased with EES [17]. Kim and colleagues demonstrated no significant divergence in MACE occurrence between first‐generation and second‐generation DESs among patients who underwent successful PCI [18]. Additionally, Yano and colleagues' study, focusing on EES deployment in small vessel lesions, revealed that while the 3‐year MACE rates did not significantly differ from those of SES, EES implantation correlated with a notable reduction in overall stent‐related events [19]. This trend was further corroborated by Yano and colleagues and Di Lorenzo and colleagues, asserting that EESs consistently improved clinical outcomes compared to SESs [20, 21].

MACE predictors play a crucial role in predicting and preventing cardiovascular events like heart attacks, strokes, and cardiovascular‐related deaths. Our study revealed additional predictors for MACE occurrence in the 5‐year study of patients, including prior history of heart failure, HTN, and prior PCI. MACE occurrence was higher in patients with lower baseline EF, a higher number of stents, and longer total stent length. Previous studies [22, 23] have shown that the risks for MACE can vary from person to person. Still, common factors that increase the risk include HTN, DM, dyslipidemia, smoking, obesity, prior PCI, family history, and CKD. A family history of CVDs, such as heart attacks or MI in first‐degree relatives, can also increase the risk of a MACE [24].

Another MACE predictor is CKD. The relationship between CKD and MACE is complex and multifactorial. The buildup of nonexcreted substances can lead to endothelial dysfunction and vascular damage, increasing the risk of a MACE [25]. However, our study did not find a significant relationship between the occurrence of MACE and CKD. Previous research has indicated that individuals with a history of PCI are more prone to developing MACE compared to those without such a history [26], possibly due to complications related to stents. It is essential to recognize that the risk of MACE can vary based on individual factors, including the extent and severity of CAD, overall health status, and adherence to prescribed treatments and lifestyle modifications [27]. By acknowledging the significance of these risk factors, healthcare professionals can take proactive measures to mitigate cardiovascular risk and enhance patient outcomes.

In 2018, a study by Nogic and colleagues demonstrated that the difference in MACE between PF‐DES and PC‐DES regarding the occurrence of MACE at the longest follow‐up was not significant; however, PF‐DES were found to be associated with a significant reduction in all‐cause death [28]. Verdoia and colleagues and Ullah and colleagues highlighted that PF‐DES significantly decreased mortality compared to PC‐DES [29, 30]. In the long term, PF‐DES, compared with PC‐DES, was associated with a lower mortality rate, but no superiority was found in short‐term mace occurrence [31]. In our cohort, PF‐DES was associated with a higher incidence of MACE (37.5%) compared to PC‐DES (10.1%), and this difference reached statistical significance (*p* = 0.002). While this finding appears notable, the extremely small number of patients receiving PF‐DES (*n* = 8) limits the reliability of this result. Given the insufficient statistical power and wide confidence intervals inherent in such a small subgroup, this observation should be considered exploratory and interpreted with caution. Larger, adequately powered studies are needed to verify this potential association.

Other findings of our study showed that the LAD was the most frequently involved coronary artery lesion in CAD based on coronary angiography (2951, 71%). A previous cross‐sectional study in Saudi Arabia cited the LAD lesion as the most common lesion in both ST‐segment and non‐ST‐segment elevation MI patients [32]. In contrast, another study in the same country found that the LCX is the most common site of coronary artery lesions, with a prevalence of 85.3%, followed by LAD lesions (82.4%) [33]. Furthermore, Type‐C lesions were the most common among different types of coronary artery lesions in our study. In contrast, a retrospective study in Turkey concluded that Type‐B and proximal lesions may be more prevalent among patients younger than 35 with acute ST‐segment elevation MI [34]. As a result, the prevalence and types of coronary lesions may differ in different populations.

This study has several limitations that should be acknowledged. First, its retrospective design inherently limits the ability to establish causal relationships and is subject to potential biases related to data completeness and accuracy. Second, the number of patients receiving nonpolymer stents was relatively small, which may have reduced the statistical power to detect significant differences in outcomes for this subgroup. Our study employed pragmatic inclusion criteria to reflect routine clinical practice, which while strengthening external validity, may have introduced heterogeneity in patient characteristics. However, residual confounding from unmeasured variables (e.g., microvascular dysfunction, complete revascularization status) remains possible. Therefore, future prospective, multicenter studies utilizing national registry systems and comprehensive databases are warranted to more accurately assess the long‐term efficacy and safety of different types of DES, particularly among patients at high risk of experiencing MACE.

## 5 Conclusions

Our findings from a 5‐year observational period indicate that the occurrence rate of MACEs among individuals undergoing PCI does not exhibit significant variations with different DES. MACEs were more frequent in individuals with a longer total stent length, a greater number of stents, a lower baseline EF, a history of heart failure, prior PCI, or hypertension.

## Author Contributions

**Hossein Koushki:** conceptualization, investigation, funding acquisition, writing – original draft, methodology, validation, visualization, software, formal analysis, data curation. **Reza Golchin Vafa:** investigation, writing – original draft, methodology, validation, visualization, software, formal analysis, data curation. **Reza Heydarzadeh:** investigation, writing – original draft, methodology, validation, visualization, formal analysis, software, data curation. **Houyar Zarifkar:** investigation, writing – original draft. **Amin Khadem Hosseini:** investigation, writing – original draft. **Houman Zarifkar:** writing – original draft, investigation. **Hourshad Zarifkar:** investigation, writing – original draft. **Alireza Azadian:** investigation. **Farhang Amiri:** investigation. **Ali Mohammadhassani:** investigation. **Mohammad Montaseri:** investigation. **Nazanin Hosseini:** investigation. **Mehrdad Sadeghi:** investigation. **Seyed Ali Hosseini:** investigation, methodology. **Seyed Alireza Mirhosseini:** writing – original draft, writing – review and editing. **Javad Kojuri:** conceptualization, investigation, supervision, project administration, writing – review and editing. All authors have read and approved the final version of the manuscript.

## Ethics Statement

This study was approved by the Ethics Committee of Shiraz University of Medical Sciences (Reference Number IR.SUMS.MED.RED.1402.409) and adhered to the ethical standards set forth by institutional and national research committees. It followed the principles of the 1964 Declaration of Helsinki and its subsequent amendments, or other equivalent ethical standards.

## Consent

Written informed consent was waived and it was obtained from all participants involved in the study. The purpose of the research was fully explained to the patients, who were assured that their personal information would remain confidential and protected by the researcher.

## Conflicts of Interest

The authors declare no conflicts of interest.

## Transparency Statement

The lead author Javad Kojuri affirms that this manuscript is an honest, accurate, and transparent account of the study being reported; that no important aspects of the study have been omitted; and that any discrepancies from the study as planned (and, if relevant, registered) have been explained.

## Supporting information


**Supplementary Table 1:** Baseline data of the study participants, mean ± standard deviation or *n* (%). **Supplementary Table 2:** Baseline angiography findings of the study participants, *n* (%). **Suppelemntary Table 3:** A summary of the baseline angioplasty characteristics of the study participants.

## Data Availability

Data are available upon reasonable request from the corresponding author. Dr. Javad Kojuri had full access to all of the data in this study and takes complete responsibility for the integrity of the data and the accuracy of the data analysis.
