# Supplementary material for: Comparative Evaluation of Incidence of Cardiovascular Events Among Different Drug‐Eluting Stent Features: A Retrospective Cohort Study
Source: Health Sci Rep. 2025 Sep 23;8(9):e71287. doi: 10.1002/hsr2.71287 (PMC12457694; doi:10.1002/hsr2.71287)
Supplement: Supplementary file 1 — Supplementary Table 1: Baseline data of the study participants, mean ± standard deviation or n (%). Supplementary Table 2: Baseline angiography findings of the study participants, n (%). Suppelemntary Table 3: A summary of the baseline angioplasty characteristics of the study participants. [file HSR2-8-e71287-s001.docx]

**Title**

**Comparative Evaluation of Incidence of Cardiovascular Events Among Different Drug-Eluting Stent Features**

**Running title: MACE in Different Drug-Eluting Stents**

**Authors**

Hossein Koushki ^1,2^, Reza Golchin Vafa ^1,3^, Reza Heydarzadeh ^1,3^, Houyar Zarifkar ^1,3^, Amin Khadem Hosseini ^1,3^, Houman Zarifkar ^1,3^, Hourshad Zarifkar ^1,3^, Alireza Azadian ^1,3^, Farhang Amiri ^1,3^, Ali Mohammadhassani ^1,3^, Mohammad Montaseri ^1,2,3^, Nazanin Hossein ^1,3^, Mehrdad Sadeghi ^3^, Seyed Ali Hosseini ^3^, Seyed Alireza Mirhosseini ^2,4^, Javad Kojuri ^1,2,3,5^

*Corresponding author: Javad Kojuri; E-mail: Kojurij@yahoo.com; [Info@kojuriclinic.com](mailto:Info@kojuriclinic.com),

Iran, Shiraz, Shahed Blv, Nyayesh medical complex, Professor Kojuri cardiology clinic

1. Shiraz University of Medical Sciences, Shiraz, Iran
2. Cardiology Department, Shiraz University of Medical Sciences, Shiraz, Iran
3. Professor Kojuri Cardiology Clinic, Niayesh St., Niayesh Medical Complex, Shiraz, Iran
4. Cardiovascular Research Center, School of Medicine, Shiraz University of Medical Sciences, Shiraz, Iran
5. Clinical Education Research Center, Shiraz University of Medical Sciences, Shiraz, Iran

**Supplementary Document**

**Supplementary Table 1.** Baseline data of the study participants, mean ± standard deviation or n (%)

| Total participants (n = 4159) |  |  |
| --- | --- | --- |
| Gender, male |  | 2904 (69.8) |
| Age, years |  | 63.7 ±12.6 |
| Body mass index, kg/m^2^ |  | 26 ± 3.7 |
| Positive family history of coronary artery disease (n, %) |  | 938 (22.6) |
| Hypertension |  | 2789 (67.1) |
| Diabetes Mellitus |  | 1449 (34.8) |
| Dyslipidemia |  | 2355 (56.6) |
| Smoking | Recent | 189 (4.5) |
|  | Current | 522 (12.6) |
| Alcohol addiction |  | 17 (0.4) |
| Opium addiction |  | 222 (5.3) |
| Prior percutaneous coronary intervention |  | 627 (15.1) |
| Prior coronary artery bypass graft |  | 227 (5.5) |
| Peripheral artery disease |  | 5 (0.1) |
| Heart failure |  | 38 (0.9) |
| Ejection fraction, % |  | 50.3 ± 10.8 |
| Chronic kidney disease |  | 62 (1.5) |
| Stable angina |  | 2400 (57.7) |
| Unstable angina |  | 610 (14.7) |
| ST-elevation myocardial infarction |  | 369 (8.9) |
| Non-ST-elevation myocardial infarction |  | 331 (8) |

**Supplementary Table 2.** Baseline angiography findings of the study participants, n (%)

| Total participants (n = 4,159) |  |  |
| --- | --- | --- |
| Number of diseased vessels | One | 2532 (60.9) |
|  | Two | 1323 (31.8) |
|  | Three | 304 (7.3) |
| Total lesions | One | 1703 (40.9) |
|  | Two | 1274 (30.6) |
|  | Three or more | 1182 (28.4) |
| Lesion territory | LAD | 2951 (71) |
|  | LCX | 1536 (36.9) |
|  | RCA | 1603 (38.5) |
| Lesion location | Proximal | 2657 (63.9) |
|  | Midpart | 2029 (48.8) |
|  | Distal | 599 (14.4) |
| Lesion type | Type A | 512 (12.3) |
|  | Type B1 | 1738 (41.8) |
|  | Type B2 | 1631 (39.2) |
|  | Type C | 1775 (42.7) |
| Involved artery | Left main | 150 (3.6) |
|  | LAD | 2782 (66.9) |
|  | Diagonal | 597 (14.4) |
|  | LCX | 1105 (26.6) |
|  | Obtuse marginal | 699 (16.8) |
|  | RCA | 1475 (35.5) |
|  | PDA | 297 (7.1) |
|  | Intermediate/anterolateral | 147 (3.5) |
|  | Posterolateral | 116 (2.8) |
|  | Ramus | 2 (<0.1) |

**Suppelemntary Table 3.** A summary of the baseline angioplasty characteristics of the study participants

| Total patients (n = 4,159) |  |  |
| --- | --- | --- |
| Total balloons (n, %) | None | 208 (5) |
|  | One | 1305 (31.4) |
|  | Two | 1115 (26.8) |
|  | Three or more | 553 (13.3) |
| Balloon technique (n, %) | Predilatation | 3404 (81.8) |
|  | Postdilatation | 2403 (57.8) |
|  | Kissing | 216 (5.2) |
| Total stents (n, %) | One | 1938 (46.6) |
|  | Two | 1373 (33) |
|  | Three or more | 848 (20.3) |
| Total stent length per patient (Mean ± SD) |  | 50.6 ± 33.2 |
| Stent diameter (Mean ± SD) |  | 3.2 ±1.1 |
| Type of stents (polymer/non-polymer) (n, %) | Polymer | 4133 (99.4) |
|  | Non-polymer | 8 (0.1) |
|  | Both | 18 (0.4) |
| Number of types of drugs eluting stents (n, %) | One | 3178 (76.4) |
|  | Two | 960 (23.1) |
|  | Three | 21 (0.5) |
| Type of drug-eluting stents (n, %) | EES | 2543 (61.6) |
|  | SES | 2434 (61.1) |
|  | ZES | 83 (2) |
|  | BES | 79 (1.9) |
|  | AES | 2 (<0.1) |
|  | PES | 2 (<0.1) |
| Stent location (n, %) | Proximal stents | 2569 (61.8) |
|  | Midpart stents | 1860 (44.7) |
|  | Distal stents | 538 (12.9) |
| Stented artery (n, %) | Left main | 150 (3.6) |
|  | LAD | 2782 (66.9) |
|  | Diagonal | 597 (14.4) |
|  | LCX | 1105 (26.6) |
|  | Obtuse marginal | 699 (16.8) |
|  | RCA | 1475 (35.5) |
|  | PDA | 297 (7.1) |
|  | Intermediate/anterolateral | 147 (3.5) |
|  | Posterolateral | 116 (2.8) |
|  | Ramus | 2 (<0.1) |
